# Outcomes of pessary fitting trials for patients with stage IV pelvic organ prolapse: a prospective study

**DOI:** 10.1007/s00192-023-05594-2

**Published:** 2023-08-05

**Authors:** Ying Zhou, Tianshu Sun, Aijing Ju, Lan Zhu

**Affiliations:** Department of Gynecology and Obstetrics, Peking Union Medical College Hospital, Chinese Academy of Medical Sciences & Peking Union Medical College, National Clinical Research Center for Obstetric & Gynecologic Diseases, No. 1, Shuaifuyuan, Dongcheng District, Beijing, 100730 China

**Keywords:** Pessary, Pelvic organ prolapse, Nonsurgical treatment

## Abstract

**Introduction and hypothesis:**

The objective was to evaluate the efficacy of pessaries in the treatment of stage IV pelvic organ prolapse (POP) and identify the influencing factors.

**Methods:**

One hundred and fifty-seven patients with stage IV symptomatic POP were admitted to the hospital for pessary fitting. A successful pessary fitting was defined as a patient fitted with a pessary at the initial fitting in whom use continued 2 weeks later. The rates of successful pessary fitting, patient satisfaction, remission of prolapse and urinary symptoms, and the occurrence of factors associated with successful pessary fitting were calculated and predictors of appropriate pessary type selection were analyzed.

**Results:**

A total of 130 patients with stage IV POP had a successful pessary fitting (82.8%). The satisfaction rate associated with the two types of pessaries was more than 90%. The success rate among patients undergoing a ring pessary fitting trial was 44.6%, and 84.3% of the patients were self-managed. Prolapse symptoms significantly improved in 90% of cases, and urinary symptoms improved in 58–93% of cases from baseline. The number of vaginal deliveries, history of hysterectomy and vaginal introitus/total vaginal length (TVL) ratio were independent risk factors associated with unsuccessful pessary fitting.

**Conclusion:**

For patients with stage IV POP, the successful fitting rate is as high as 80% or more. More vaginal deliveries, a history of hysterectomy, and a larger vaginal introitus/TVL ratio (ratio  >0.6) were predictors of unsuccessful pessary fitting.

## Introduction

Pelvic organ prolapse (POP) is the main pelvic floor disorder in adult women. Chinese epidemiological survey results show that the prevalence of symptomatic POP is 9.6% in adult women and approximately 50% in postmenopausal women [1, 2]; it is not fatal but seriously affects quality of life. Treatment for POP includes surgical or nonsurgical options. As a non-invasive treatment, pessaries can provide immediate and significant relief of symptoms. Given the effectiveness and safety of pessaries, a survey conducted by the American Urological Association reported that nearly two thirds of physicians choose pessaries as a first-line treatment for POP. Chinese guidelines for POP recommended pessaries as a first-line treatment for POP as well [2].

Pessaries can be categorized into two types: support and space occupying. Ring pessaries and Gellhorn pessaries are the most common support and space-occupying pessaries respectively. Patients with ring pessaries are more receptive and self-managed; therefore, ring pessaries represent the preferred method of treatment. The pelvic organ prolapse quantitation (POP-Q) scores were applied to describe prolapse, and grade IV prolapse is defined as a complete eversion of the total length of the lower genital tract with the distal portion of the prolapse protruded to at least (total vaginal length (TVL) −2) cm (i.e., quantitation value  ≥  + [TVL −2] cm), which is the most severe case of prolapse. Ring pessaries are generally considered for use in patients with stage I and II symptomatic POP. Gellhorn pessaries are often used to treat stage III and IV prolapse [3]. Although studies have demonstrated that pessaries can be used in patients with various stages of prolapse, there are limited clinical data on patients with the most severe stage IV prolapse. Studies involving large amounts of data on the use of ring pessaries in patients with stage IV prolapse are even rarer. Therefore, this prospective study was designed to investigate ring pessary fitting outcomes in stage IV POP patients who received the treatment with good acceptance while also evaluating short-term efficacy, identifying the factors that influence the fitting outcome and pessary type selection and predict the success rate of pessary treatment, and clarifying whether ring pessaries can be the first choice for patients with stage IV POP.

## Materials and methods

### Study participants

A total of 157 patients with stage IV symptomatic prolapse were admitted to the Department of Obstetrics and Gynecology of Peking Union Medical College Hospital between November 2013 and July 2021. All patients were recommended to participate in the pessary fitting trial first, and those who refused or failed fitting with any pessary were offered surgical management. Approval was obtained from the Research Ethical Committee of the Peking Union Medical College Hospital (ZS-2164).

The exclusion criteria for pessary treatment were as follows:

1. Patients with vaginitis or vaginal ulceration
2. Patients requiring surgery for other gynecological diseases in addition to prolapse
3. Patients with a history of silicone allergy
4. Patients who were not able to care for the pessary themselves and did not have a family member who was willing and able to provide weekly pessary care
5. Patients with mental illness

Written informed consent was obtained from the study participants.

Clinical staging was performed according to the POP-Q, and TVL and internal vaginal introitus width were measured. Baseline clinical data, medical history information, and physical examination results were collected from patients. At baseline, patients were asked the following questions about prolapse symptoms:

1. Do you see or feel a bulge in your vagina?
2. Do you feel pelvic pressure?

The following questions about urinary symptoms were asked:

1. Do you leak urine when you cough, laugh, sneeze, or exercise?
2. Do you leak urine when you have the urge to empty your bladder?
3. Do you have to strain to empty your bladder or have difficulty emptying your bladder?
4. Do you need to insert your fingers into your vagina to void urine?

Replies of “never” or “rarely” were recorded as “no,” whereas replies of “sometimes,” “usually,” or “always” were recorded as “yes.” Physical examinations and pessary administrations were performed on all patients by experienced urogynecologists.

The initial fitting usually involved applying a ring pessary with support, followed by a Gellhorn pessary for patients with failure of ring pessary fitting. In general, the largest pessary that was comfortable for the patient was used. The diameter of both pessaries ranged from 51 to 76 mm. Patients who were comfortably fitted with a pessary were asked to perform the Valsalva maneuver, ambulate and void over the course of half an hour while they were at the office. If the pessary remained comfortable, the patients were educated on how to manage the pessary. Briefly, we recommended that patients remove and clean their pessary at bedtime and reinsert it the next morning at least once a week. The type and size of the pessary inserted were recorded. The fitting was considered unsuccessful if a patient was unable to be fitted with any pessary, and the reasons for failure were recorded.

The successfully fitted patients were followed up within 2 weeks. The pessary was removed and cleaned at the visit to check for vaginal damage. The patient was asked about symptoms such as vaginal bleeding, abnormal vaginal discharge, pain or discomfort, and de novo urinary incontinence. When asked the same questions again, the answers were recorded in the same way. A symptom was “resolved” if the baseline reply was “yes” and the 2-week reply was “no.” A symptom was “persistent” if the baseline reply was “yes” and the 2-week reply was “yes.” A symptom was “de novo” if the baseline reply was “no” and the 2-week reply was “yes.” Patient satisfaction was assessed using the five-point Likert scale of the Patient's Global Impression of Change (PGI-C) questionnaire, which involved the following question: Are you satisfied with pessary treatment? As an answer, five choices were available as follows: 5) very satisfied, 4) somewhat satisfied, 3) not very dissatisfied, 2) somewhat dissatisfied, and 1) very dissatisfied.

After a 2-week follow-up, patients who reported discomfort or expulsion were offered another size or type of pessary if they wanted to continue with the trial. They then returned after 2 weeks for as many as three times and were evaluated as described above on each occasion. Successful pessary fitting was defined as a patient who was fitted with a pessary at the initial fitting in whom use continued 2 weeks later. The patients who discontinued pessary use could return to discuss surgical options if desired. The number of fitting attempts and the subjective reasons for discontinuation were carefully recorded.

### Statistics

Means and standard deviations were calculated for continuous variables. Nonparametric tests were used to compare continuous variables between groups. Continuous corrected Chi-squared tests were used to compare categorical variables. Values with *p* ≤ 0.05 were considered statistically significant. Multivariate logistic regression was used to analyze possible risk factors. Statistical analysis was performed using SPSS v.24.0 (IBM Corp, Armonk, NY, USA).

## Results

**Fig. 1:**
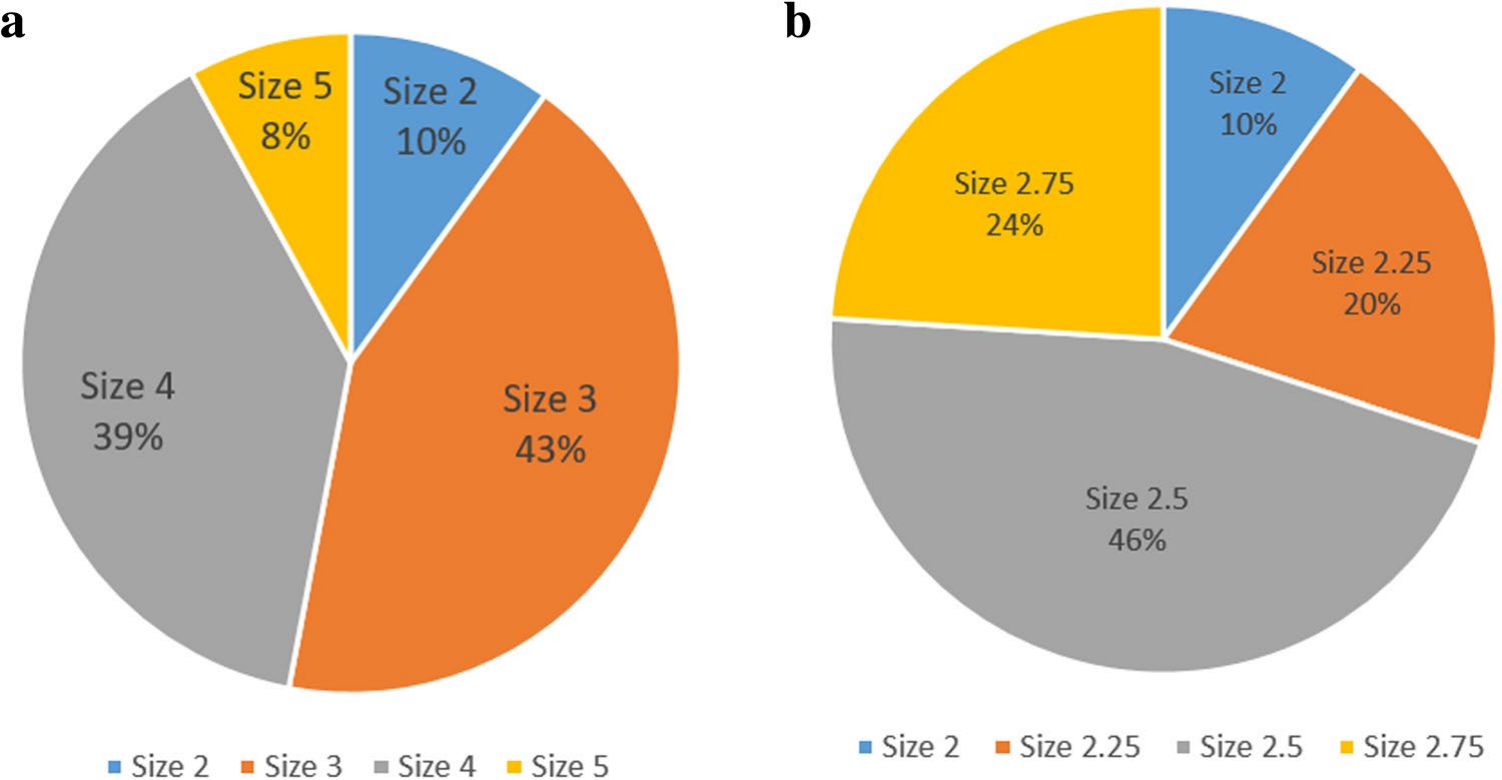
Pessary size selection. **a** The proportion of ring pessaries with support after the fitting trial. **b** The proportion of Gellhorn pessaries after the fitting trial

**Fig. 2:**
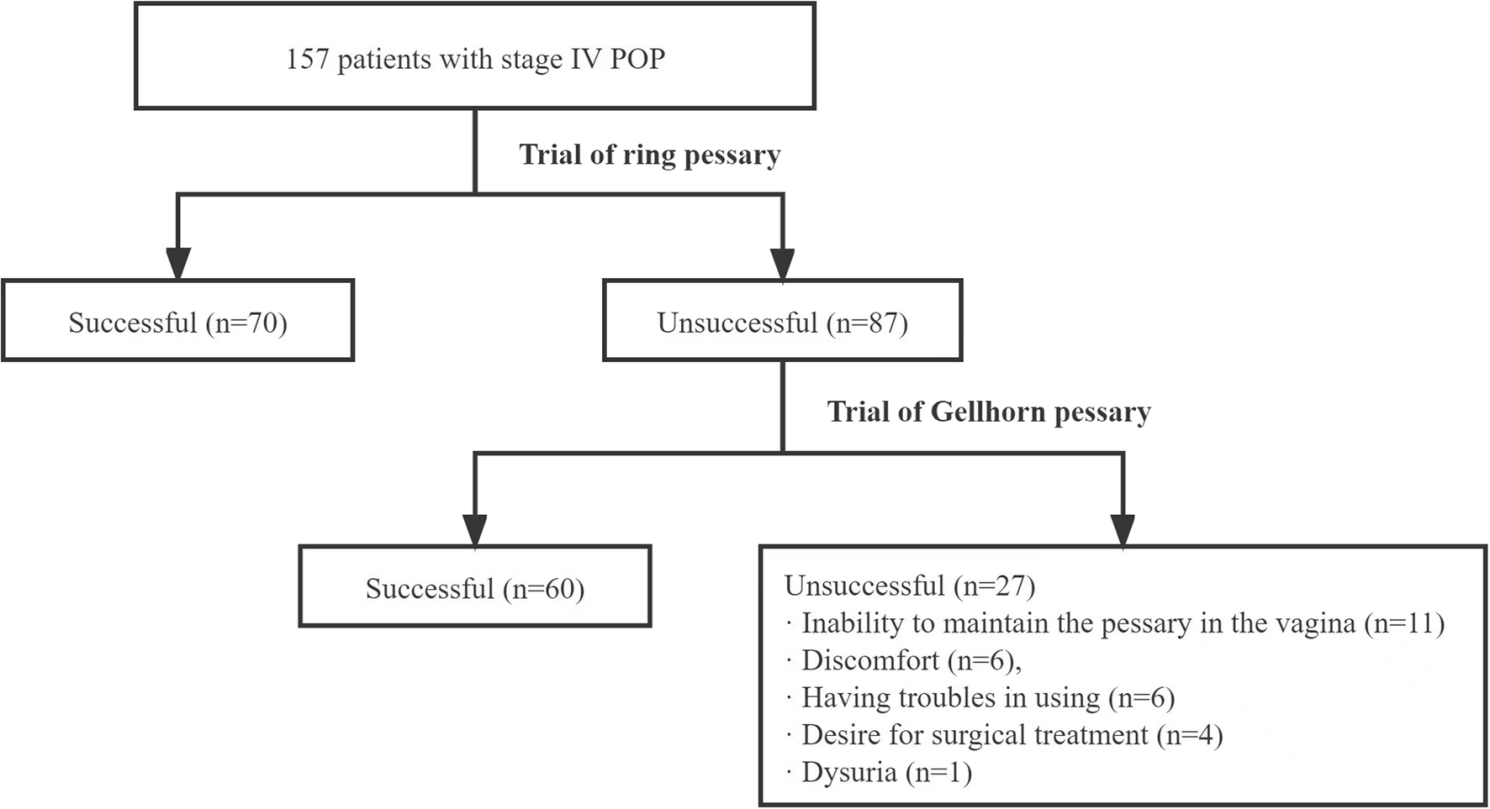
Study flowchart (the numbers of patients who were successfully and unsuccessfully fitted and the reasons for unsuccessful pessary fitting)

Pessary trials were performed on 157 patients with stage IV prolapse, and almost all patients were postmenopausal (154, 98.1%). Seventy patients (44.6%, 70 out of 157) were successfully fitted with the ring pessary, and those in whom a ring pessary failed were fitted with a Gellhorn pessary. Sixty patients (69.0%, 60 out of 87) were successful among them, for an overall success rate of 82.8% (130 out of 157). Four different sizes of ring pessaries (2, 3, 4, 5) and Gellhorn pessaries (2, 2.25, 2.5 and 2.75) were used. Size 3 (diameter = 64 mm) and size 4 (diameter = 70 mm) ring pessaries and size 2.5 (diameter = 64 mm) Gellhorn pessaries were most commonly used (Fig. 1). Fourteen patients were unable to be fitted with the pessary at the initial trial, and 13 women stopped pessary fitting at the 2-week visit, comprising 27 (17.2%) patients in whom the trial failed. The reasons for unsuccessful fittings of either pessary type were “inability to maintain the pessary in the vagina” (11 out of 27), “discomfort” (6 out of 27), “having troubles in using” (6 out of 27), “desire for surgical treatment” (4 out of 27), and “dysuria” (1 out of 27; Fig. 2).

There were no significant differences in age or body mass index (BMI) between the two groups of patients who had successful and unsuccessful pessary fitting. Univariate analysis of patient characteristics in the successful and unsuccessful groups (Table 1) showed that the parameters of number of deliveries, history of hysterectomy, posterior wall as the predominant compartment of prolapse, and vaginal width/TVL (ratio > 0.6) were significantly associated with failed pessary fitting (*p* < 0.05). All predictors associated with fitting failure (*p* < 0.05) were included in the multivariate regression analysis, and the results (Table 2) showed that a history of previous hysterectomy, vaginal width/TVL (ratio >0.6), and the number of vaginal deliveries were independent predictors of failed fitting. Most prominently, 35 out of 157 patients (22.3%) had undergone previous hysterectomy, of whom 13 (37.1%) had a failed pessary fitting.

**Table 1:** Univariate analysis of the predictors for successful and unsuccessful pessary fitting

	Successful (*n* = 130)	Unsuccessful (*n* = 27)	*p*
Age	70.0 ± 8.2	70.7 ± 12.6	0.779
BMI	24.7 ± 3.2	24.8 ± 3.0	0.79
<25	76	17	0.329
≥25	54	10	
Vaginal delivery times	2.4 ± 1.2	3.1 ± 1.4	0.009
Menopause	128	26	1.00
Largest baby ≥4 kg	18	4	0.959
History of hysterectomy	22	13	0.001
Predominant prolapse compartment			
Anterior vaginal wall	103	22	0.055
Uterine	107	15	0.526
Posterior vaginal wall	36	13	0.024
TVL	8.0 ± 1.1	7.6 ± 1.2	0.082
<7.5 cm	27	11	0.866
≥7.5 cm	103	16	
Vaginal introitus width	4.5 ± 0.6	4.7 ± 0.8	0.060
<5 cm	81	15	0.380
≥5 cm	49	12	
GH	5.4 ± 0.89	5.6 ± 0.84	0.617
<6 cm	76	14	0.206
≥6 cm	54	13	
GH/TVL	0.69 ± 0.14	0.74 ± 0.12	0.062
Vaginal width/TVL	0.56 ± 0.09	0.64 ± 0.14	0.001
>0.6	35	16	0.001

*BMI* body mass index, *GH* genital hiatus, *TVL* total vaginal length

**Table 2:** Multivariate analysis of the predictors of successful and unsuccessful pessary fitting

	β	Significance	Upper limit	Lower limit
Vaginal delivery times	0.501	0.019	1.088	2.505
History of hysterectomy	1.527	0.003	1.669	12.710
Predominant prolapse compartment in posterior vaginal wall	0.501	0.106	0.840	6.216
Vaginal introitus width/TVL >0.6	1.155	0.023	1.172	8.594

*TVL* total vaginal length

Of the 130 patients who were fitted with the pessaries successfully, 70 were successful with ring pessaries, and 60 were successful with Gellhorn pessaries. Further analysis of the two groups (Table 3) revealed that patients with high BMI, more history of previous hysterectomy, posterior wall-predominant prolapse, and vaginal width  ≥5 cm were more likely to be successful in fitting the Gellhorn pessary, with statistically significant differences between the two groups (*p* < 0.05). All predictors associated with the type of pessary (*p* < 0.05) were included in the multivariate regression analysis, and the results showed (Table 4) that a history of previous hysterectomy and vaginal width  ≥5 cm were independent risk factors for the choice of pessary type.

**Table 3:** Univariate analysis of successful fitting of ring and Gellhorn pessaries

	Ring (*n* = 70)	Gellhorn (*n* = 60)	*p*
Age	70.4 ± 8.6	69.5 ± 7.7	0.517
BMI	24.1 ± 2.7	25.3 ± 3.6	0.028
<25	47	29	0.030
≥25	23	31	
Vaginal delivery times	2.2 ± 1.1	2.6 ± 1.3	0.151
Menopause	69	59	0.450
Largest baby ≥4 kg	14	4	0.053
History of pelvic surgery	7	15	0.034
Predominant prolapse compartment			
Anterior vaginal wall	57	46	0.505
Uterine	58	49	0.859
Posterior vaginal wall	14	22	0.034
TVL	8.0 ± 1.1	8.0 ± 1.0	0.859
<7.5 cm	13	14	0.505
≥7.5 cm	57	46	
Vaginal introitus width	4.4 ± 0.5	4.6 ± 0.7	0.063
<5 cm	52	29	0.002
≥5 cm	18	31	
GH	5.3 ± 0.9	5.5 ± 0.9	0.224
<6 cm	43	33	0.458
≥6 cm	27	27	
GH/TVL	0.55 ± 0.10	0.57 ± 0.09	0.210
IH/TVL	0.68 ± 0.17	0.69 ± 0.09	0.716
>0.6	14	21	0.055

*BMI* body mass index, *TVL* total vaginal length, *GH* genital hiatus, *IH *introitus hiatus

**Table 4:** Multivariate analysis of successful and unsuccessful fitting of two types of pessaries

	β	Significance	Upper limit	Lower limit
BMI	0.121	0.079	0.986	1.292
History of hysterectomy	1.347	0.016	1.283	11.535
Predominant prolapse compartment in posterior vaginal wall	0.528	0.279	0.652	4.413
Vaginal introitus width ≥5 cm	1.004	0.022	1.155	6.448

*BMI* body mass index

At the return visit within 2 weeks of pessary fitting, the patient satisfaction rates (PGI-C score 5) for treatment with the ring pessary and the Gellhorn pessary were 94.3% (66 out of 70) and 90% (54 out of 60) respectively, with no significant difference (*p* > 0.05). However, compared with 58.3% of patients (35 out of 60) fitted with Gellhorn pessaries who could care for themselves, 84.3% (59 out of 70) of patients with ring pessaries could care for themselves, which was significantly different (*p* = 0.001). Before treatment, each patient had at least one symptom. Prolapse symptoms were commonly pelvic pressure (71.3%, 112 out of 157) and increased discharge (16.5%, 26 out of 157). Urinary symptoms were commonly voiding difficulty (66.9%, 105 out of 157), urge urinary incontinence (48.4%, 76 out of 157), stress urinary incontinence (43.3%, 68 out of 157), and splinting to void (41.4%, 65 out of 157; Table 5). Prolapse and most urinary symptoms were significantly improved in patients who were successfully fitted with both types of pessaries (*p* < 0.05, Table 6). Regarding proportions of patients who experienced improvements in particular symptoms, a total of 89.6% (86 out of 96) with pelvic pressure, 60.9% (14 out of 23) with increased vaginal discharge, 93.3% (84 out of 90) with voiding difficulty, 93.0% (53 out of 57) with splinting to void, and 71.0% (44 out of 62) with urge urinary incontinence symptoms were affected. Symptoms of stress urinary incontinence improved in 58.8% (30 out of 51), but 16 patients (20.3%) had new-onset incontinence, indicating insignificant symptom improvement. Increased vaginal discharge was the most common complication, with an incidence of 34.6% (45 out of 130). Seven patients with ring pessaries and 5 patients with Gellhorn pessaries (9.2%, 12 out of 130) reported uncomfortable sensations. A total of 16.2% of patients (11 out of 68) had new urgency to urinate, and 1 patient (1 out of 40, 2.5%) with a Gellhorn pessary had new difficulty urinating. There was no significant difference between the ring and Gellhorn pessaries in terms of improvement of prolapse and urinary symptoms (*p* > 0.05, Table 7).

**Table 5:** Pre-treatment symptoms of pelvic organ prolapse patients

Symptom		Number	Percentage
Prolapse symptom	Pelvic pressure	112	71.3
	Increased discharge	26	16.5
Urinary symptom	Voiding difficulty	105	66.9
	Urge urinary incontinence	76	48.4
	Stress urinary incontinence	68	43.3
	Splinting to void	65	41.4

**Table 6:** Improvement of prolapse and urinary symptoms in patients with successful trial pessaries at the 2-week follow-up

Symptom	Pretreatment	2-week follow-up		Symptom remission rate (%)	*p*
		Yes	No		
Pressure	Yes	10	86	89.6	0.000
	No	0	34		
Increased vaginal discharge	Yes	9	14	60.9	0.017
	No	31	76		
Voiding dysfunction	Yes	6	84	93.3	0.000
	No	1	39		
Urgency incontinence	Yes	18	44	71.0	0.000
	No	11	57		
Stress incontinence	Yes	21	30	58.8	0.055
	No	16	63		
Vaginal splinting	Yes	4	53	93.0	0.000
	No	0	73

**Table 7:** Improvement in prolapse and urinary symptoms in patients who were successfully fitted with two types of pessaries at the 2-week follow-up

Symptom	Pretreatment	2-week follow-up				*p*
		Yes		No		
		Ring	Gellhorn	Ring	Gellhorn	
Pressure	Yes	4	6	45	41	0.686
	No	0	0	21	13	0.171
Increased vaginal discharge	Yes	6	3	7	7	0.722
	No	19	12	38	38	0.288
Voiding dysfunction	Yes	2	4	46	38	0.307
	No	0	1	22	17	0.202
Urgency incontinence	Yes	13	5	21	23	0.079
	No	6	5	30	27	0.907
Stress incontinence	Yes	13	8	15	15	0.400
	No	9	7	33	30	0.782
Vaginal splinting	Yes	2	2	34	19	0.894
	No	0	0	34	39	–

## Discussion

In this study, the success rate in the pessary trial was 82.8% (130 out of 157) in patients with stage IV POP, and the success rate in the ring pessary trial was 44.6% (70 out of 157). For those in whom the ring pessary trial failed, the success rate of the Gellhorn pessary trial was 69.0% (60 out of 87). A history of hysterectomy, increased number of deliveries, and vaginal width/TVL (ratio >0.6) were independent risk factors for unsuccessful fitting. Our study further demonstrated that pessaries can be used in patients with different stages of POP. Even in patients with severe prolapse, pessaries can be selected as the first-line treatment. In addition, we found that the ring pessary was not only suitable for patients with mild prolapse but also achieved a satisfactory success fitting rate and equivalent clinical efficacy in patients with stage IV prolapse. As the ring pessary is easier to self-manage and more acceptable to patients, it is recommended that the ring pessary be the first choice of treatment in clinical practice, and the Gellhorn pessary can be used as an alternative if the ring pessary trial fails.

Pessaries are divided into supporting and space-filling types. Many domestic and international studies have reported success rates ranging from 41 to 96% for pessary fitting [4–9]. The large variation was due to the different timing of successful fitting as defined by each study. The success rate after 2 weeks of initial fitting was 58–88% [10–13], and it has been confirmed that pessaries can be applied in patients with various stages of prolapse. The stage of prolapse was not a determinant of fitting trial success [14, 15], but several studies have indicated that the stage of prolapse was associated with fitting failure [16, 17]. This study, performed by a dedicated treatment team led by a specialist at Peking Union Medical College Hospital for pessary placement, further demonstrated that pessaries can have a similarly high success rate, even for patients with stage IV prolapse. At the same time, patient satisfaction with pessary treatment was as high as 90%. Approximately 90% of prolapse and dysuria and 70% of urinary urgency symptoms were notably relieved, and urinary incontinence symptoms were relieved in more than half of the patients. This greatly enhances the confidence of physicians and patients in choosing pessaries as the first-line treatment decision for POP. Our results also suggest that having the same physician perform the operation might improve the success rate of pessary fitting considerably.

This study also demonstrated that the ring pessary was suitable not only for patients with stages II and III symptomatic prolapse but also for patients with stage IV prolapse. Our study found that the ring pessary was comparable with the Gellhorn pessary in terms of relief of prolapse, urinary symptoms, and satisfaction with pessary therapy, but patients with the ring pessary were more receptive and self-managed (84% vs 58%). Thus, it was again confirmed that the nonsurgical pessary should be the preferred treatment for POP, including stage IV. The trial process for pessaries follows the recommendation that the ring pessary should be the first choice of pessary treatment for all POP, including stage IV POP, and that the Gellhorn pessary may be an option after failure of the ring pessary.

In this study, a history of hysterectomy and a high vaginal width/TVL ratio were found to be independent factors influencing trial success in patients with stage IV prolapse. Previous studies reported the same conclusion that a history of hysterectomy significantly decreased the success rate of pessary fitting [4–7, 10], suggesting that a short TVL (≤7.5 cm,  ≤7.3 cm,  ≤7 cm,  <6 cm) affected the success rate of the trial, and some gave specific predictive figures [11–13, 18]. The presumed cause may be the narrowing and stiffness of the vaginal cuff and the shortening of the vaginal length that can result from surgery. Some studies have also suggested an effect of genital hiatus (GH), the ratio of GH to TVL and vaginal introitus width measured by POP-Q on the success rate of the trial, but the conclusions were not uniform [8, 9, 12, 13, 19, 20]. However, we found that the vagina was relatively longer in patients with stage IV prolapse (8 cm in the success group vs 7.6 cm in the failure group), and it was speculated that the vaginal cuff was affected by the surgery and may have contributed to the fitting failure. This study also found that GH, the ratio of GH to TVL, and wide vaginal introitus width did not affect successful fitting in patients with stage IV prolapse, whereas the high vaginal introitus width/TVL ratio was an independent factor influencing fitting success. We speculate that this may be related to the design characteristics and working principle of the pessary. Compared with TVL, the middle and lower vaginal segments are relatively wide, and a wider vaginal introitus requires the selection of a pessary with a larger transverse diameter; at the same time, the relative lack of vaginal length or the relative narrowing of the vaginal cuff limits the selection of the pessary size and makes it easier for the pessary to fall and cause discomfort, which eventually leads to fitting failure.

Further analysis of pessary selection in this study revealed that patients with a history of hysterectomy and vaginal width  ≥5 cm failed more often to be fitted with a ring pessary but were successfully fitted with a Gellhorn pessary; this suggested that patients with a history of hysterectomy and wide vaginal width (transverse vaginal diameter) might be more prone to failure with a ring pessary and more suited to a Gellhorn pessary, which was consistent with the findings of previously published articles [13, 17, 21]. This difference in suitability appears to be related to the design features and working principles of the different types of pessaries [22]. The supporting ring pessary mainly forms a supporting structure under the posterior vault and symphysis pubis and is located in the middle of the vagina to support the prolapsed tissue. A wide vaginal introitus may suggest a decrease in the attachment of the bulbocavernosus muscle to the perineal body, and dilatation of the vaginal introitus may affect the maintenance of the position of the ring pessary, resulting in a drop in the pessary. In patients with a history of hysterectomy, the narrow, stiff upper and middle vaginal segments caused by surgery can only accommodate fairly small pessaries, resulting in a relatively large width of the vaginal introitus and failure to fit the ring pessary. In contrast, the Gellhorn pessary has special structural features, such as a short stem and concave disk, which can interact with the vaginal wall at the vaginal cuff and provide greater suction and more support to the prolapsed tissue, compensating for the influence of the vaginal cuff on the ring pessary while relying less on the pelvic floor tissue structure of the vaginal introitus. However, in clinical practice, the fitting trial is still started with the ring pessary because it is more convenient for patients to operate.

The strength of this study was that it was a prospective study that included a relatively large sample size of patients with stage IV prolapse given the current literature. In addition, all data were obtained from the same treatment center, and all patients were examined by only one physician, ensuring consistency in measurements such as physical examination POP-Q scores and vaginal width. The main limitation of the study was the limited selection of pessaries, with only ring pessaries for the support type and only Gellhorn pessaries for the space-filling type, which may have slightly reduced the success rate of the pessary fitting trial.

## Conclusion

We found 82.8% success with the pessary fitting trial for women with stage IV POP, a treatment satisfaction rate of over 90%, and a significant improvement in prolapse and most urinary symptoms. The success rate with the ring pessary was 44.6%, and the treatment outcome did not differ from that achieved with the Gellhorn pessary, so it could be the first choice for nonsurgical treatment of all POP, including stage IV. The parameters of the number of deliveries, history of previous hysterectomy, and vaginal width/TVL (ratio >0.6) were independent risk factors for fitting failure. Patients with a history of previous hysterectomy and vaginal width  ≥5 cm are better candidates for a Gellhorn pessary trial.

## Data Availability

Data available on request from the authors.

## Declarations

### Submission

This research has not been published previously, and it is not under consideration for publication elsewhere.
